# Diagnostic value of fine-needle aspiration biopsy for breast mass: a systematic review and meta-analysis

**DOI:** 10.1186/1471-2407-12-41

**Published:** 2012-01-25

**Authors:** Ying-Hua Yu, Wei Wei, Jian-Lun Liu

**Affiliations:** 1 Departmant of Breast Surgery of Guangxi Cancer Hospital & Affiliated Cancer Hospital of Guangxi Medical University, Nanning 530021, Guangxi, P.R.China

## Abstract

**Background:**

Fine-needle aspiration biopsy (FNAB) of the breast is a minimally invasive yet maximally diagnostic method. However, the clinical use of FNAB has been questioned. The purpose of our study was to establish the overall value of FNAC in the diagnosis of breast lesions.

**Methods:**

After a review and quality assessment of 46 studies, sensitivity, specificity and other measures of accuracy of FNAB for evaluating breast lesions were pooled using random-effects models. Summary receiver operating characteristic curves were used to summarize overall accuracy. The sensitivity and specificity for the studies data (included unsatisfactory samples) and underestimation rate of unsatisfactory samples were also calculated.

**Results:**

The summary estimates for FNAB in diagnosis of breast carcinoma were as follows (unsatisfactory samples was temporarily exluded): sensitivity, 0.927 (95% confidence interval [CI], 0.921 to 0.933); specificity, 0.948 (95% CI, 0.943 to 0.952); positive likelihood ratio, 25.72 (95% CI, 17.35 to 28.13); negative likelihood ratio, 0.08 (95% CI, 0.06 to 0.11); diagnostic odds ratio, 429.73 (95% CI, 241.75 to 763.87); The pooled sensitivity and specificity for 11 studies, which reported unsatisfactory samples (unsatisfactory samples was considered to be positive in this classification) were 0.920 (95% CI, 0.906 to 0.933) and 0.768 (95% CI, 0.751 to 0.784) respectively. The pooled proportion of unsatisfactory samples that were subsequently upgraded to various grade cancers was 27.5% (95% CI, 0.221 to 0.296).

**Conclusions:**

FNAB is an accurate biopsy for evaluating breast malignancy if rigorous criteria are used. With regard to unsatisfactory samples, futher invasive procedures are required in order to minimize the chance of a missed diagnosis of breast cancer.

## Introduction

Palpable breast mass is a common problem in female patients. The diagnostic delays of breast cancer occur due to the generally low index of suspicion. The traditional diagnosis mode of breast mass is excisional biopsy, which gives a precise diagnosis but may yield a benign pathological result in most cases.

Fine-needle aspiration biopsy (FNAB) of the breast is a minimally invasive diagnostic method, often obviating an open biopsy [1]. It is cheaper to perform and its results can be available within a shorter time. However, the role of FNAB has been challenged of late by better overall results attained by core biopsies. Core biopsy is definitely a robust and reliable diagnostic modality, but carries disadvantages in terms of a longer turn-around due to the tissue processing time, and patient discomfort during the procedure. FNAB has some advantages over core-needle biopsy in that it use a smaller needle and thus has a lower probability of causing hematoma and other rare complications, such as pneumothorax [2,3]

With the introduction of stereotactic and ultrasonographically (US) guided methods for nonpalpable lesions, fine-needle aspiration biopsy (FNAB) have been used more widely in the evaluation of nonpalpable breast lesions [4-6]. Furthermore, the triple-diagnostic method (consisting of clinical evaluation, mammography and FNAB) gives a precise diagnosis and reduces the risk of missed diagnosis of breast cancer to < 1% [7].

However, the clinical use of FNAB has been questioned because of the variability in results reported [8] In addition, It is also possible that no cells are harvested making cytological analysis impossible. Many institutes in the United Kingdom, the United States and Canada have now abandoned FNA for diagnosis of breast lesions. Nevertheless, it continues to be used in other institutes in these countries, as well as in Greece, Italy, Australia and Japan, and in developing countries such as India, Pakistan, Nigeria, Mexico and Thailand. Up to now, there was no meta-analysis to establish the overall value of FNAB for the diagnostic breast cancer. The purpose of our study was to establish the overall value of FNAC in the diagnosis of breast lesions.

## Materials and methods

### Search strategy and study selection

We searched MEDLINE (1966 to 2010), EMBASE (1970 to 2010), the Cochrane Central Register of Controlled Trials (CENTRAL), database of Health Technology Assessments on The Cochrane Library issue 2, 2010 and the China Biological Medicine Database (CBM-disc, 1979 to 2010), VIP Chinese Journals Database (1968 to 2010), China National Knowledge Infrastructure Whole Article Database (CNKI, 1994 to 2010). We also searched the trials registers of Cochrane Breast Cancer Group and the WHO International Clinical Trials Registry at http://www.who.int/ictrp/en/ for ongoing and recently completed trials. All searches were up to date as of December 2010. The search terms used were "breast neoplasms", "fine-needle aspiration biopsy", "Sensitivity and Specificity" and "accuracy". In addition, related keywords and their synonyms were included in our search strategy and reference lists were scanned for additional publications. In order to form a highly sensitive search strategy, there were no restrictions on publication status, or study design. Although no language restrictions were imposed initially, for the full-text review and final analysis, our resources only permitted the review of articles published in the English and Chinese language. Letters, conference abstracts and grey literature to the journal editors were excluded because of the limited data presented.

From the studies obtained in the above search, only those meeting the following criteria were qualified for subsequent analyses: Chinese and English languages, any study designed with at least thirty patients, reporting all-comers populations with suspicious breast lesion after clinic screening. The index biopsy method is fine-needle aspiration biopsy (FNAB) with or without image-guide. Two reviewers (Yinghua Yu, Wei Wei) selected eligible studies independently. Discrepancies were solved by discussion.

### Data extraction and quality assessment

Data extraction was performed independently by two reviewers (Yinghua Yu, Wei Wei)). The two reviewers were blinded to publication details, and all extracted data had to be agreed upon by them. Data retrieved from the reports including study design, participant characteristics, lesion size, FNAB procedures, outcomes measurement, publication year, and methodological quality. The numbers of true-positive, false-positive, false-negative, and true-negative results are displayed for each study in Table 1.

**Table 1:** Summary of including studies (Insufficient Samples Excluded in test result)

study	year	reference standard	test result				patients (number)	QUADAS score
					
			TP	FP	FN	TN		
Walker et al.[33]	1998	his	91	0	0	19	110	13
Kanchanabat et al.[11]	2000	his/imag and clin	8	8	0	26	42	10
Pisano et al.[12]	2001	his/imag and clin	59	18	17	183	277	11
Lumachi et al.[34]	1999	his	55	0	2	5	62	10
Farshid et al.[35]	2008	his/imag and clin	91	120	2	880	1093	11
Dennison et al.[36]	2003	his/imag and clin	95	0	10	38	143	10
Gent et al.[37]	1986	his	39	7	1	109	156	13
Wanebo et al.[38]	1983	his	123	8	1	102	234	13
Apesteguia et al.[39]	1997	his/imag and clin	2	2	0	107	111	11
Manheimer et al.[40]	1977	his	79	2	11	35	127	11
Janet et al.[41]	2010	his/imag and clin	11	0	4	135	150	9
Drew et al.[42]	1999	his/imag and clin	102	4	27	130	263	8
Masood et al.[43]	1991	his	17	0	3	71	91	8
Ishikawa et al.[44]	2007	his	138	4	4	178	324	12
Harvey et al.[45]	1996	his/imag and clin	14	37	13	32	96	8
Leifland et al.[46]	2003	his	353	11	52	27	443	11
Okamoto et al.[47]	1998	his/imag and clin	5	1	1	125	132	11
Rubin et al.[48]	1997	his/imag and clin	34	4	0	27	65	10
Sauer et al.[32]	2003	his/imag and clin	360	21	47	321	749	11
Li et al.[49]	2008	his	129	1	22	108	260	9
Wang et al.[50]	2010	his	110	2	7	55	174	13
Yang et al.[51]	2010	his	79	0	4	259	342	11
Liu et al.[52]	2000	his	205	1	12	194	412	10
Zhao et al.[53]	2009	his	218	10	6	241	475	11
Zhan et al.[54]	2007	his	32	2	0	44	78	9
Gao et al.[55]	2005	his	169	0	27	85	281	11
Lu et al.[56]	2010	his	102	0	33	48	183	10
Liu et al.[57]	2010	his	5	1	1	125	132	11
Zhang et al.[58]	2008	his	39	6	4	53	102	11
Zhang et al.[59]	2006	his	21	2	3	31	57	9
Tang et al.[60]	1987	his	144	2	13	144	303	11
Wang et al.[61]	1995	his	25	0	2	22	49	10
Ma et al.[62]	2010	his	56	0	2	99	157	8
Wang et al.[63]	2010	his	96	12	4	498	610	8
Wang et al.[64]	1981	his	442	43	60	479	1024	10
Wei et al.[65]	2007	his	91	4	2	78	175	12
Tao et al.[66]	2004	his	655	5	14	2027	2701	12
Wang et al.[67]	2005	his	99	5	0	207	311	11
Chen et al.[68]	2010	his	66	1	9	176	252	8
Zeng et al.[69]	1999	his	94	1	2	66	163	11
Jia et al.[70]	1986	his	49	0	1	50	100	9
Ma et al.[71]	2010	his	68	0	4	291	363	10
Huang et al.[72]	2003	his	305	31	54	436	826	11
Zhang et al.[73]	1996	his	21	1	2	40	64	9
Yu et al.[74]	2006	his	1620	116	33	359	2128	11
Jiang et al.[75]	2008	his	66	1	9	176	252	11

His: histopathology; imag and clin:imaging and clinical follow-up; TP:true positive; FP: false positive; FN:false negative; TN: true negative.

We assessed the methodological quality of the studies using guidelines published by the quality assessment for studies of diagnostic accuracy (QUADAS) tool [9] [i.e. appraisal by use of empirical evidence, expert opinion, and formal consensus to assess the quality of primary studies of diagnostic accuracy]. In addition, for each study the following characteristics of study design were also retrieved: (1) needle gauge (21 gauge vs other size gauge); (2) guidance systems (with ultrasound or stereotactic guidance vs without image guidance); (3) prospective data collection, and (4) geographic location; (5) reference standard (histopathology only or not). If no data on the above criteria were reported in the primary studies, we requested the information from the authors. If the authors did not respond to our letters, the "unknown" items were treated as "no."

### Statistical analysis

We used standard methods recommended for meta-analyses of diagnostic test evaluations [10]. Analyses were performed using RevMan5 software (the Cochrane Information Management System (IMS)), STATA version 10.0 (STATA Corporation, TX, USA) and Meta-DiSc (for Windows; XI Cochrane Colloquium; Barcelona, Spain).

We categorized the cytological results according to The National Cancer Institute recommendation for the diagnosis of breast aspiration cytology:

• Cl = unsatisfactory.

• C2 = cells present all benign; no suspicious features.

• C3 = cells suspicious but probably benign.

• C4 = cells suspicious but probably malignant.

• C5 = Definitely malignant.

This category' is used when the degree of diagnostic certainty is such that the pathologist would be happy for the patient to undergo major breast surgery on the basis of the cytology sample alone.

We classified the results of FNAB as positive (including class C3, C4, C5) and negative (C2) adapted from Burapa Kanchanabat [11] and Etta D. Pisano [12]. C1 was temporarily exluded from this classification. Our reason for this classification is that solid masses with a FNAB result of intermediate classification (C3, C4,) require other diagnostic procedures to minimized the underestimation. The following measures of test accuracy were computed for each study: sensitivity; specificity; diagnostic odds ratio (DOR); positive likelihood ratio (PLR) and negative likelihood ratio (NLR). As for unsatisfactory samples (C1), it includes disfigured cellular morphology that cannot be interpreted and fewer than five epithelial cell groups. Disputes about unsatisfactory samples were among these studies on account of following therapeutic decisions. In most of the studies, unsatisfactory samples were excluded from analysis while considered to be positive in a study [12]. Since unsatisfactory samples played important roles on therapeutic decisions, we also assessed the sensitivity and specificity for the studies which reported unsatisfactory samples (unsatisfactory samples considered to be positive in our classification) and underestimation rate of unsatisfactory samples.

The analysis was based on a summary receiver operating characteristic (SROC) curve [13,14]. Sensitivity and specificity for the single test threshold identified for each study were used to plot an SROC curve. Random-effects model was used to calculate the average sensitivity, specificity, and the other measures across studies [15].

The term heterogeneity when used in relation to meta-analyses refers to the degree of variability in results across studies. We used the X2 and Fisher exact tests to detect statistically significant heterogeneity. To assess the effects of QUADAS scores on the diagnostic ability of FNAB, we included it as covariates in univariate meta-regression analysis (inverse variance weighted). We also analyzed the effects of other covariates on DOR (ie, publication year, guidance systems, 21 or other size needle gauge, different reference standard, prospective data collection, and different geographic location). The relative DOR (RDOR) was calculated according to standard methods to analyze the change in diagnostic precision in the study per unit increase in the covariate [16,17]. Since publication bias is of concern for meta-analyses of diagnostic studies, we tested for the potential presence of this bias using funnel plots [18].

## Results

After independent review, 59 publications dealing FNAB for the diagnosis of breast cancer were considered to be eligible for inclusion in the analysis. Of these publications, eight studies [19-26] were excluded because FNAB was performed only in breast cancer patients, two studies [27,28] were excluded because they evaluated the role of FNAB in diagnosis of axillary lymph nodes, two studies [29,30] were excluded because they did not allow the calculation of sensitivity or specificity or underestimate rate, one studies [31] were excluded because its data had been included as a part of the latest study [32]. Subsequently, 46 studies [11,12,32-75] including 7207 patients with breast cancer or suspicious features and 9435 patients with benign mass were available for analysis, and the clinical characteristics of these studies, along with QUADAS scores, are outlined in Table 1. In addition, we derived relative data about unsatisfactory samples from 11 [11,12,32,33,35,37-39,44,46,50] studies in table 2.

**Table 2:** Test result of studies (Insufficient Samples Classified as Positive)

study	test result				Patients number
		
	TP	FP	FN	TN	
Walker et al.[33]	95	11	0	19	125
Kanchanabat et al.[11]	8	13	0	26	47
Pisano et al.[12]	80	149	17	183	429
Farshid et al.[35]	92	173	2	880	1147
Gent et al.[37]	39	38	1	109	187
Wanebo et al.[38]	135	18	1	102	256
Apesteguia et al.[39]	2	36	0	107	145
Ishikawa et al.[44]	156	44	4	178	382
Leifland et al.[46]	406	37	52	27	522
Sauer et al.[32]	382	82	47	321	832
Wang et al.[50]	119	5	7	55	186

TP:true positive; FP: false positive; FN:false negative; TN: true negative.

### Study characteristics and quality of studies

As shown in Table 1 and Table 3 the average sample size of the included studies was 362 patients (range, 42 to 2128). Twenty-night studies were performed in Asia; Seventeen studies were conducted in NA (Canada and USA) and Europe. With the exception of one study which was multicenter [12], the others were single center. Most studies were case series (41 retrospective, 5 prospective).

**Table 3:** characteristics of studies

study	study design prospective	geographic location Asia	21 needle gauge	Apply imaging guidance systems	histopathology standard only
Walker et al.[33]	yes	no	yes	no	Yes
Kanchanabat et al.[11]	yes	yes	no	no	no
Pisano et al.[12]	yes	no	no	yes	no
Lumachi et al.[34]	no	no	not mention	yes	yes
Farshid et al.[35]	yes	no	not mention	no	no
Dennison et al.[36]	yes	no	yes	no	no
Gent et al.[37]	no	no	no	yes	yes
Wanebo et al.[38]	no	no	no	yes	yes
Apesteguia et al.[39]	no	no	no	yes	no
Manheimer et al.[40]	no	no	no	no	yes
Janet et al.[41]	no	yes	no	no	no
Drew et al.[42]	no	no	not mention	no	no
Masood et al.[43]	no	no	not mention	no	yes
Ishikawa et al.[44]	no	yes	no	no	yes
Harvey et al.[45]	no	no	not mention	yes	no
Leifland et al.[46]	no	no	yes	yes	yes
Okamoto et al.[47]	no	yes	yes	yes	no
Rubin et al.[48]	no	no	not mention	no	no
Sauer et al.[32]	no	no	not mention	yes	no
Li et al.[49]	no	yes	not mention	no	yes
Wang et al.[50]	no	yes	not mention	no	yes
Yang et al.[51]	no	yes	not mention	no	yes
Liu et al.[52]	no	yes	not mention	no	yes
Zhao et al.[53]	no	yes	yes	no	yes
Zhan et al.[54]	no	yes	yes	no	yes
Gao et al.[55]	no	yes	yes	no	yes
Lu et al.[56]	no	yes	no	yes	yes
Liu et al.[57]	no	yes	yes	yes	yes
Zhang et al.[58]	no	yes	no	no	yes
Zhang et al.[59]	no	yes	yes	yes	yes
Tang et al.[60]	no	yes	yes	no	yes
Wang et al.[61]	no	yes	no	yes	yes
Ma et al.[62]	no	yes	no	no	yes
Wang et al.[63]	no	yes	no	no	yes
Wang et al.[64]	no	yes	yes	no	yes
Wei et al.[65]	no	yes	yes	no	yes
Tao et al.[66]	no	yes	no	no	yes
Wang et al.[67]	no	yes	yes	no	yes
Chen et al.[68]	no	yes	yes	no	yes
Zeng et al.[69]	no	yes	no	no	yes
Jia et al.[70]	no	yes	yes	no	yes
Ma et al.[71]	no	yes	no	no	yes
Huang et al.[72]	no	yes	yes	no	yes
Zhang et al.[73]	no	yes	yes	no	yes
Yu et al.[74]	no	yes	yes	no	yes
Jiang et al.[75]	no	yes	yes	no	yes

The age of patients ranged from 26 to 87 years. Risk factors for cancer in the selected population, i.e., family history, genetic predisposition, menopausal status, and/or prior high risk lesions, were rarely reported. Needle size has varied from 20 to 25 gauge in these studies, 15 studies used 21-gauge size while the others used 20, 22, 23, 25 size or didn't mentioned it. Four studies used stereotactic guidance systems; five studies used ultrasound systems; four studies used stereotactic or ultrasound guidance systems according to the breast mass; the rest studies performed FNAB without any kind of imaged guidance systems. Two outcome measurements were adopted by these studies. As histopathologic results from surgical biopsy was regarded as gold standard in this field, patients with malignant lesions, cells suspicious, or when requested, subsequently underwent open surgery in all these studies. In case of benign lesions (breast cysts and a benign cytological result from low-risk patients), clinical and imaged follow-up at least 6 months was adopted by some studies while most of the studies still have involved comparison of FNAB benign findings with histopathologic results from core-needle biopsy or open surgical biopsy.

We assessed the quality of the studies using QUADAS. Out of 14 QUADAS items, item 1 (spectrum composition) and item 2 (selection criteria) are about the variability of studies, item 8 (index test execution), 9 (reference standard execution) and 13 (uninterpretable test results) are about the quality of reporting, the rest of the items are about the bias of studies. If criterion was fulfilled, the item was signed "yes"; if not, the item was signed "no"; if study did not report clearly and we could not request the information from the authors, the item was signed "unknown". As shown in Table 4, item 1 and 2 were 91% and 100% fulfilled by studies respectively; item 8, 9, 13 were 80%, 100%, 24%; in the rest of the items assessed bias reached a high level, except item 11 (reference standard review bias) and 14 (withdrawals).

**Table 4:** methodological quality of 46 studies

	QUADAS item													
	
	1	2	3	4	5	6	7	8	9	10	11	12	13	14
Yes(n)	42	46	46	40	46	35	46	37	46	46	7	39	11	13
No(n)	4	0	0	0	0	11	0	5	0	0	0	0	0	3
Unknown(n)	0	0	0	6	0	0	0	4	0	0	39	7	35	30
Yes(%)	91	100	100	87	100	76	100	80	100	100	15	85	24	28

n: number of studies

### Diagnostic accuracy

**Figure 1:**
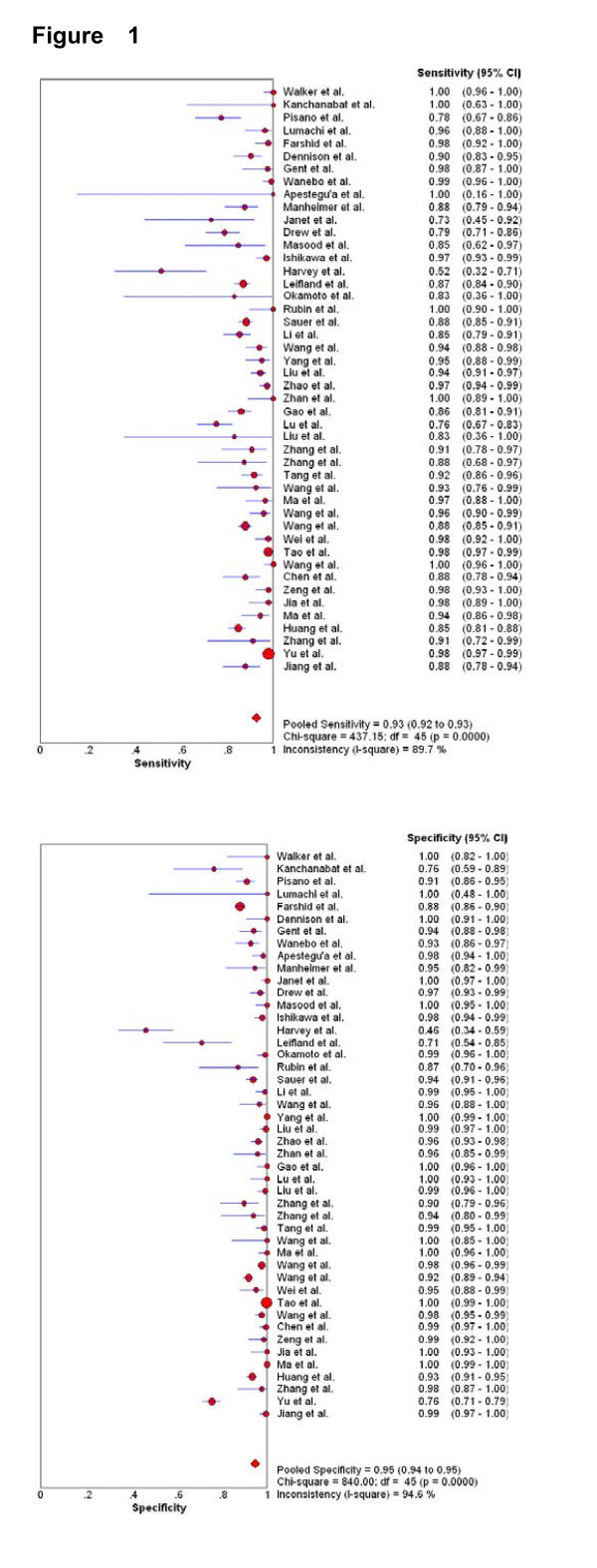
**Forest plot of estimates of sensitivity and specificity for FNAB (C1 was temporarily exluded) in the diagnosis of breast cancer**. The point estimates of sensitivity and specificity from each study are shown as solid circles. Error bars are 95% confidence intervals. The authors' names indicate the studies.

Figure 1 shows the forest plot of sensitivity and specificity for FNAB in the diagnosis of breast cancer (C1 was temporarily exluded). The sensitivity ranged from 0.52 to 1.00 (mean, 0.927; 95% confidence interval [CI], 0.921 to 0.933), and specificity ranged from 0.46 to 1.00 (mean, 0.948; 95% CI, 0.943 to 0.952). We also noted that PLR was 25.72 (95% CI, 17.35 to 28.13), NLR was 0.08 (95% CI, 0.06 to 0.11), and DOR was 429.73 (95% CI, 241.75 to 763.87). X2 values of sensitivity, specificity, PLR, NLR, and DOR were 437.15 (p < 0.001), 840.00 (p < 0.001), 653.52 (p < 0.001), 464.80 (p < 0.001), and 396.91 (p < 0.001), respectively, with all indicating a significant heterogeneity between studies.

**Figure 2:**
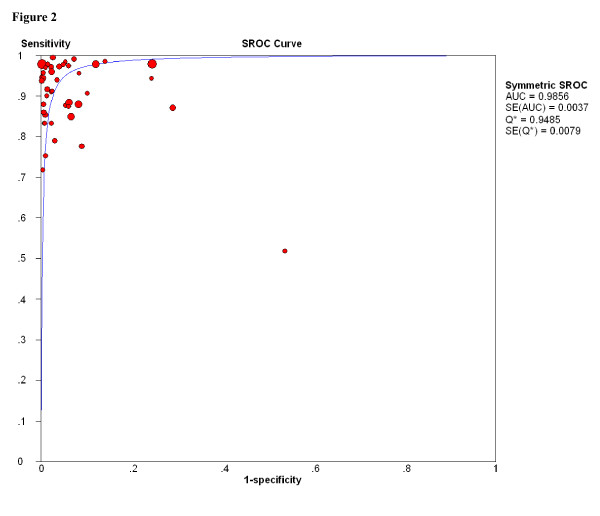
**SROC curves for FANB (C1 was temporarily exluded)**. Each study is represented by each solid circle in the meta-analysis. The size of the solid circle indicates the size of each study. SROC curves summarize the overall diagnostic accuracy.

The SROC curve presents a global summary of test performance, and shows the tradeoff between sensitivity and specificity. A graph of the SROC curve for the biopsy results of FNAB (C1 was temporarily exluded) showing true-positive rates vs false-positive rates from individual studies is shown in Figure 2. Our data showed that the SROC curve is positioned near the desirable upper left corner of the SROC curve, and that the maximum joint sensitivity and specificity (ie, the Q-value) was 0.948; while the area under the curve (AUC) was 0.986, indicating a high level of overall accuracy.

**Figure 3:**
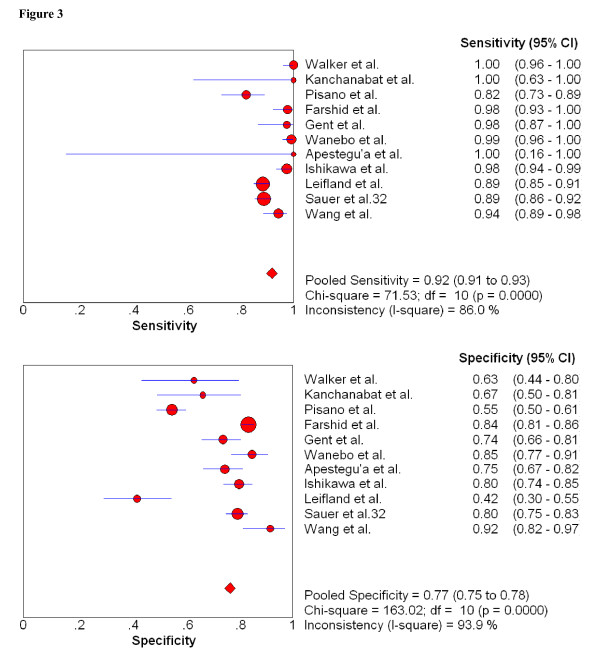
**Forest plot of estimates of sensitivity and specificity for FNAB in 11 studies which reported insufficient samples**. Insufficient samples considered to be positive.

**Figure 4:**
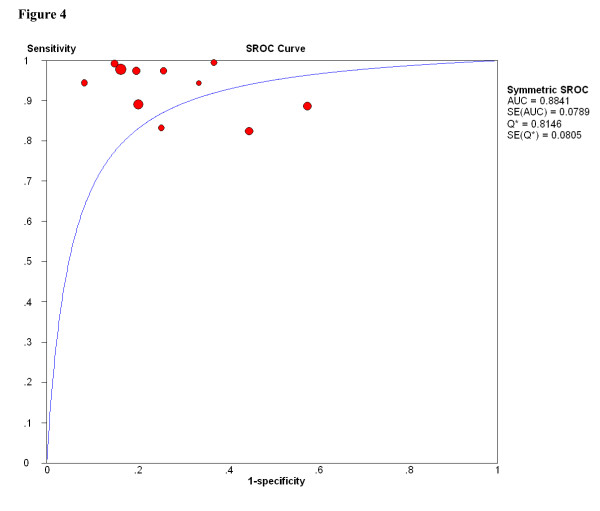
**SROC curves for FANB of 11 studies which reported insufficient samples (insufficient samples was considered to be positive)**. Each study is represented by each solid circle in the meta-analysis. The size of the solid circle indicates the size of each study. SROC curves summarize the overall diagnostic accuracy.

Since unsatisfactory samples played important roles on therapeutic decisions, we assessed the pooled sensitivity and specificity for FNAB in the diagnosis of breast cancer in these 11 studies [11,12,32,33,35,37-39,44,46,50], which reported unsatisfactory samples (C1 was considered to be positive in this classification). As Figure 3 shows, the pooled sensitivity and specificity for this group were 0.920 (95% CI, 0.906 to 0.933; X2 = 71.53, p < 0.001) and 0.768 (95% CI, 0.751 to 0.784; X2 = 163.02, p < 0.001) respectively. This graph of the SROC curve is shown in Figure 4. The maximum joint sensitivity and specificity was 0.815; while the area under the curve (AUC) was 0.884. In addition, we calculated and displayed the underestimation rates of unsatisfactory samples between FNAB and histopathologic standard in table 5. The pooled proportion of these unsatisfactory samples that were subsequently upgraded to various grade cancers was 27.5% (95% CI, 0.221 to 0.296), X2 values was 159.85 (p < 0.001). This p-values also indicated significant heterogeneity between studies.

**Table 5:** Summary underestimation rate of unsatisfactory samples using meta-analysis

	n	a	b	pooled under-estimation rate	95%CI	X^2^	P-value
unsatisfactory Samples	11	140	545	25.7%	0.221-0.296	159.85	p < 0.001

n = number of studies; a = number of breast cancer or Low grade cancers; b = total number of unsatisfactory Samples;

### Multiple regression analysis and publication bias

We used meta-regression to assess the different aspects among 46 studies: with or without 21-gauge, with or without imaging guidance systems, standard reference adopted histopathology only, located in Asia, prospective designs and QUADAS scores. Quality scoring was done by the use of QUADAS [76], in which a score of 1 was given when a criterion was fulfilled, 0 if a criterion was unclear, and -1 if the criterion was not achieved. As was shown in table 1, the studies were with relative high quality if its score was more than ten. These scores were used in the meta-regression analysis to assess the effect of study quality on the RDOR of FNAB in the diagnosis of breast mass. The table 6 showed that excepted two aspects (with or without imaging guidance systems, standard reference adopted histopathology only), the rest aspects mentioned above did not substantially affect the diagnostic accuracy as their differences did not reach statistical significance (p > 0.05).

**Table 6:** meta-regression of the effects of six different studies' aspects on diagnosis value of FNAB

Covariates	Number of studies	Coefficient	RDOR	95%CI	P-value
QUADAS ≥ 10	34	0.747	2.11	0.60-7.47	0.2389
21-gauge	19	-0.705	0.49	0.16-1.53	0.2149
imaging guidance systems	13	-1.383	0.25	0.07-0.95	0.0417
histopathology only	35	1.705	5.5	1.04-29.08	0.0451
prospective	5	0.74	2.1	0.25-17.36	0.4829
Asia	31	0.433	1.56	0.33-7.43	0.5696

**Figure 5:**
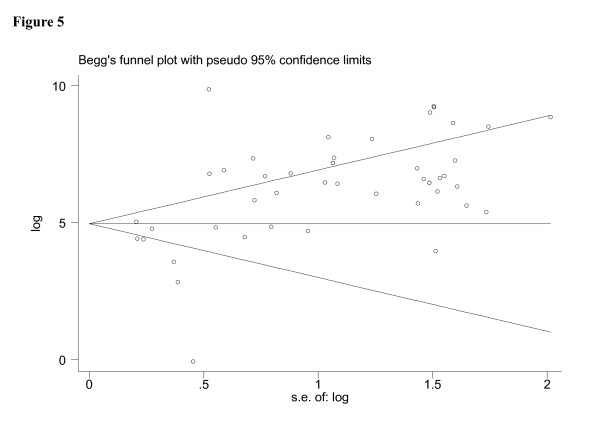
**Funnel graph for the assessment of potential publication bias in FNAB**. The funnel graph plots the log of the DOR against the SE of the log of the DOR (an indicator of sample size). Forty-six circle represents forty-six studies in the meta-analysis. The line in the centre indicates the summary diagnostic odds ratio.

The funnel plots for publication bias (Figure 5) showed large asymmetry. These results indicated a potential for publication bias.

## Discussion

Our current meta-analysis presented the high level diagnostic accuracy of Fine-needle aspiration biopsy (FNAB). In our first classification (C1 was temporarily exluded as most studies did.), the sensitivity rate was 92.7% and the specified rate was nearly 94.8%. The SROC curve showed the maximum joint sensitivity and specificity (i.e. the Q-value) was 0.948; while the area under the curve (AUC) was 0.986, presenting excellent level of overall accuracy.

The DOR is a single indicator of test accuracy [77] that combines the data from sensitivity and specificity into a single number. The DOR of a test is the ratio of the odds of positive test results in the patient with disease relative to the odds of positive test results in the patient without disease. The value of a DOR ranges from 0 to infinity, with higher values indicating better discriminatory test performance (i.e. higher accuracy). A DOR of 1.0 indicates that a test does not discriminate between patients with the disorder and those without it. In the present meta-analysis, we have found that the mean DOR was 429.73, also indicating a high level of overall accuracy.

Since the SROC curve and the DOR are not easy to interpret and use in clinical practice, and since likelihood ratios are considered to be more clinically meaningful [78,79], we also presented both PLR and NLR as our measures of diagnostic accuracy. Likelihood ratios of > 10 or < 0.1 generate large and often conclusive shifts from pre-test to post-test probability (indicating high accuracy) [79]. In our first classification, PLR value of 25.72 suggests that patients with various grade cancers have an approximately 26-fold higher chance of being FNAB result-positive compared with patients with benign breast lesion. This high probability would be considered high enough to begin surgical treatment or other therapy. On the other hand, NLR was found to be 0.08 in our current meta-analysis. If the FNAB result was negative, the probability that this patient has breast carcinoma is approximately 8%.

It should be emphasized that we used the approach of Burapa Kanchanabat [11] and Etta D. Pisano [12] for evaluating the diagnostic performance of FNAB (1. unsatisfactory samples was temporarily excluded; 2. unsatisfactory samples was classified as positive). In our first classification, unsatisfactory samples (C1) was exluded as most studies did. In our second classification, Inadequate cytological material have to be interpreted as "positive". Because treating the unsatisfactory result as a negative outcome is a poor policy that has the potential to cause harm to patients and delay the diagnosis of breast cancer. On the purpose of minimizing the chance of a missed diagnosis of breast cancer, certain discrepancies between FNAB and open biopsy (e.g. cytological results including C3, C4, C5 on FNAB and atypical hyperplasia or various grades cancer on open biopsy) were considered as agreements and needed further management. The reclassified agreement rate is therefore a clinically relevant and pragmatic estimate for the accordance between FNAB and actual disease status.

Breast cancer was present in certain proportion of the inadequate FNAB specimens. Since unsatisfactory samples (C1) played important roles in influencing diagnostic accuracy of FNAB, we also assessed the pooled sensitivity and specificity for FNAB in the other classification (unsatisfactory samples were regarded as positive) and the underestimation rate of unsatisfactory samples. This pooled sensitivity (92.7%) was similar with the sensitivity (92.0%) that mentioned above in our first classification (unsatisfactory samples was exluded) while the pooled specificity (76.8%) was lower than the specificity (94.8%) above. This change may be due to the underestimation rate of inadequate samples which was currently assessed in our study. This pooled unsatisfactory samples' underestimate rate was 27.5% which was higher than the value (8.5%) reported by H.C.Lee [80]. However, we included more recent related studies and more patients than H.C.Lee did. Our underestimate rate indicated that 27.5% of the patients with a diagnosis of inadequate samples for cytological analysis will prove to have various grades breast cancer. This rate was not low enough to rule out breast cancer. So, in most of these cases, an additional managemant such as core biopsies or surgical procedure will then be necessary.

On the whole, the quality of the included studies is higher than median level according to QUADAS. Many studies did not reach item 11 (reference standard review bias), 13 (uninterpretable test results) or 14 (withdrawals). According to QUADAS items and studies' detail analysis, most studies did not mention blinding results interpreted, uninterpretable test results or explained withdrawals which did not match item 11, 13 and 14. These bias would affect the analysis of accuracy of FNAB.

An exploration of the reasons for heterogeneity rather than the computation of a single summary measure was an important goal of meta-analysis [81]. In our meta-analysis, QUADAS scores were used in the meta-regression analysis to assess the effect of study quality on RDOR. We did not observe that the studies with relatively higher quality (QUADAS score of ≥10) had better test performances than those with lower quality.

Although we found a significant heterogeneity for sensitivity, specificity, PLR, NLR and DOR among the studies analyzed, meta-regression results showed that 3 different aspects among 46 studies (such as needle size, study locations and prospective/retrospective designs) didn't reach statistical significance, indicating that these aspects did not substantially affect diagnostic accuracy. On the other hand, 2 different aspects such as guidance systems (with ultrasound or stereotactic guidance vs without imaging guidance) and reference standard (histopathology only or not) affect the diagnostic accuracy in great part. These may be due to the following reasons. First, fine-needle aspiration biopsy without imaging guidance is not suitable for patients with ill-defined masses because the aspiration cannot be done at the exact position and the cytological result may not represent the true nature of the mass. In other words, breast lesions could be definitely localized by imaging guidance then FNAB could be done. FNAB with imaging guidance system can make a favorite diagnosic accuracy. Second, there were the combined two standard methods adopted by some included studies, surgery biopsy for suspicious lesion and imaging or clinic follow-up for benign cytological result from low-risk patients. Moreover, the time of follow-up is different from each other (range from 6-24 months). As a result, misclassification may occur easier in this two different reference standard situation than in that histopathologic is the only reference standard.

Apart for having a comprehensive search strategy, our study assessed the FNAB diagnosis accuracy in all directions, such as sensitivity, specificity, PLR, NLR, DOR, SROC curve and AUC. In addition, we assessed the influence of unsatisfactory samples on FNAB diagnosis accuracy. Heterogeneity and potential publication bias were also explored in accordance with published guidelines. However, our systematic review had some limitations. Only including English and Chinese language studies and the lack of conference abstracts, letters to editors might have led to publication bias.

## Conclusion

On the whole, our current evidence shows that fine-needle aspiration biopsy (FNAB) is an accurate biopsy for evaluating breast malignancy if rigorous criteria are used. With high sensitivity and specificity, most benign and malignant breast lesions can be reliably diagnosed by FNAB. FNAB may provide a favorable screening method and permit an improvement of treatment planning. With the introduction of imaging guided methods for percutaneous sampling of nonpalpable lesions, FNAB can be used more widely in the evaluation of breast lesions

However, as unsatisfactory samples' underestimate rate (27.5%) is not low enough to rule out malignant, the result of C1 for cytological analysis in FNAB warrants futher invasive procedures including core biopsies or open surgical biopsy in order to minimize the chance of missed diagnosis of breast cancer. Fine needle aspiration continues to be an acceptable and reliable procedure for the preoperative diagnosis of breast lesions, particularly in developing countries

## Abbreviations

FNAB: Fine-needle aspiration biopsy; His: histopathology; Imag and clin: clinical and imaging follow-up; AUC: area under the curve; CI: confidence interval; DOR: diagnostic odds ratio; IFN: interferon; NLR: negative likelihood ratio; PLR: positive likelihood ratio; QUADAS: quality assessment for studies of diagnostic accuracy; RDOR: relative diagnostic odds ratio; ROC: receiver operating characteristic; SROC: summary receiver operating characteristic.

## Competing interests

The authors declare that they have no competing interests.

## Authors' contributions

Jian Lun Liu conceived of the study. Ying Hua Yu was in charged of its design and coordination. Ying Hua Yu and Wei Wei performed the statistical collection and analysis. Ying Hua Yu drafted the manuscript. All authors read and approved the final manuscript.

## Pre-publication history

The pre-publication history for this paper can be accessed here:

http://www.biomedcentral.com/1471-2407/12/41/prepub
